# Adrenomedullin and Adrenomedullin-Targeted Therapy As Treatment Strategies Relevant for Sepsis

**DOI:** 10.3389/fimmu.2018.00292

**Published:** 2018-02-19

**Authors:** Christopher Geven, Matthijs Kox, Peter Pickkers

**Affiliations:** ^1^Department of Intensive Care Medicine, Radboud University Medical Center, Nijmegen, Netherlands; ^2^Radboud Center for Infectious Diseases (RCI), Radboud University Medical Center, Nijmegen, Netherlands

**Keywords:** adrenomedullin, sepsis, treatment, antibodies, vascular barrier function, septic shock

## Abstract

Sepsis remains a major medical challenge, for which, apart from improvements in supportive care, treatment has not relevantly changed over the last few decades. Vasodilation and vascular leakage play a pivotal role in the development of septic shock, with vascular leakage being caused by disrupted endothelial integrity. Adrenomedullin (ADM), a free circulating peptide involved in regulation of endothelial barrier function and vascular tone, is implicated in the pathophysiology of sepsis. ADM levels are increased during sepsis, and correlate with extent of vasodilation, as well as with disease severity and mortality. *In vitro* and preclinical *in vivo* data show that administration of ADM exerts anti-inflammatory, antimicrobial, and protective effects on endothelial barrier function during sepsis, but other work suggests that it may also decrease blood pressure, which could be detrimental for patients with septic shock. Work has been carried out to negate ADMs putative negative effects, while preserving or even potentiating its beneficial actions. Preclinical studies have demonstrated that the use of antibodies that bind to the N-terminus of ADM results in an overall increase of circulating ADM levels and improves sepsis outcome. Similar beneficial effects were obtained using coadministration of ADM and ADM-binding protein-1. It is hypothesized that the mechanism behind the beneficial effects of ADM binding involves prolongation of its half-life and a shift of ADM from the interstitium to the circulation. This in turn results in increased ADM activity in the blood compartment, where it exerts beneficial endothelial barrier-stabilizing effects, whereas its detrimental vasodilatory effects in the interstitium are reduced. Up till now, *in vivo* data on ADM-targeted treatments in humans are lacking; however, the first study in septic patients with an N-terminus antibody (Adrecizumab) is currently being conducted.

## Introduction

Sepsis remains a major health problem in the twenty-first century, with an increasing incidence and high mortality in intensive care units worldwide (1, 2). Sepsis is an inflammatory syndrome in which a dysregulated host response to infection results in life-threatening organ dysfunction (3). Its most severe form, septic shock, is defined by increased lactate levels and vasopressor requirement to maintain sufficient blood pressure and organ perfusion, despite adequate fluid resuscitation (3). The sepsis syndrome is characterized by a very complex, multilayered pathogenesis, that involves many harmful and protective pathways (4, 5). The vascular endothelium is a protective barrier involved in the maintenance of vessel integrity that controls diffusion of molecules between the intravascular and interstitial space. Endothelial dysfunction is one of the major hallmarks of sepsis (6). The profound inflammatory response observed in sepsis plays a pivotal role in this phenomenon, which is accompanied by endothelial cell (EC) death and loss of barrier integrity (5, 7, 8). Underlying processes of loss of barrier integrity include increased actomyosin contraction (also known as “stress-fiber formation”) in response to phosphorylation of myosin light chains by myosin light chain kinase (MLCK) (9). Loss of barrier integrity leads to extravascular accumulation of fluids and molecules, causing edema, a decreased blood pressure and subsequent organ failure. A considerable percentage of mortality occurs in the early phase of sepsis, when multiorgan failure develops despite supportive therapies. Although the general knowledge of the pathophysiology of sepsis has improved, this has not translated to a single effective adjuvant therapy. The lack of clinical trials that show a therapeutic benefit may partially be explained by large patient heterogeneity, but also because of the complexity of the pathophysiology (8, 10). Thus, there is still an urgent and unmet need for new therapeutic options, and interventions that may improve the endothelial barrier function and vascular tone are an attractive category (11). A key hormone involved in regulation of the endothelium barrier and vascular tone is adrenomedullin (ADM). In this review, we describe the general vascular properties of ADM and provide an overview of the current understanding of the role of ADM in sepsis and septic shock. Furthermore, we discuss the potential of ADM and ADM-targeted treatments for sepsis patients.

## Adrenomedullin

Adrenomedullin was first discovered in human pheochromocytoma tissue in 1993 (12). Although ADMs initially discovered effects were vasodilation and blood pressure lowering effects (12–14), later work demonstrated that ADM exerts a multitude of biological actions, in both health and disease (15, 16). ADM is a 52 amino acid peptide belonging to the calcitonin gene-related peptide family (17). In humans, the gene encoding ADM is located on chromosome 11 and consists of 4 exons and 3 introns (18). The gene is transcribed into a pre-messenger RNA (mRNA) molecule, containing four exons and three introns. The removal of all introns results in the formation of a mature mRNA molecule (form A) which is eventually translated and processed into ADM as detailed below. However, if the third intron of this pre-mRNA molecule is not removed, this results in the formation of a longer mRNA molecule (form B). Due to the presence of a stop codon in this intron, a smaller prohormone is produced that does not result in the production of ADM (19). It remains unknown which factors regulate the splicing of this third intron and whether this is altered during sepsis. Translation of the form A mRNA molecule leads to a 185 amino acid long preprohormone (prepro-ADM) that undergoes a multistep cleavage process. First, a 21-residue N-terminal signaling peptide is cleaved of prepro-ADM, generating a 164 amino acid pro-ADM peptide. Next, pro-ADM is cleaved into pro-ADM N-terminal 20 peptide (PAMP) (20–22), midregional pro-ADM (MR-proADM) (23), adrenotensin (24), and a glycine-extended 53-amino acid peptide, the latter of which is subsequently converted to the 52 amino acid mature ADM by enzymatic amidation to an extent, which may vary depending on the pathology and other factors (25). Besides ADM, several of the other cleavage products are also vasoactive (i.e., PAMP exerts vasodilatory effects, whereas adrenotensin is vasoconstrictive). ADM is widely expressed in virtually all human tissues. The highest concentrations of the peptide were found in the adrenal medullae, cardiac atria, and lungs (26, 27), whereas the highest concentrations of ADM mRNA were measured in the lungs, cardiac atria, aorta, and mesenteric arteries (28). Many cells are capable of producing ADM, including ECs, vascular smooth muscle cells (VSMCs), monocytes, renal parenchymal cells, and macrophages (29–35). ADM exerts its effects by ligation of receptor complexes consisting of the calcitonin receptor-like receptor (CRLR) combined with a specific receptor activity-modifying protein (RAMP) (36, 37). The ADM1 and ADM2 receptors consist of the CRLR combined with RAMP2 and RAMP3, respectively, whereas the combination of CRLR and RAMP1 forms the CGRP (calcitonin gene-related protein) receptor. Most functional studies do not specify which receptor is specifically activated and are therefore referred to as “ADM receptors.” Analogous to the ubiquitous expression of the ADM peptide, ADM receptors have also been detected in various tissues and organs, including blood vessels, skeletal muscles, heart, lungs, and nerve tissue (38–41). On a cellular level, ADM receptors are expressed on many different cell types, including ECs, VSMCs, cardiomyocytes, macrophages, and dendritic cells (33, 42–44). Interaction of ADM with its receptor occurs through its C-terminal moiety (45), and the N-terminal part of ADM is thought to be only of minor importance for its agonist function (46). Circulating ADM has a half-life of approximately 22 min (47) and is rapidly degraded from its N-terminus by proteases (48–50). Moreover, it has been reported that the three CRLR/RAMP receptors are internalized upon stimulation together with ADM, and thus function as clearance receptors (51, 52). On a more organ-specific level, the lungs appear to be involved as a site of clearance (53, 54).

## The Role of ADM in the Regulation of Blood Pressure

**Figure 1:**
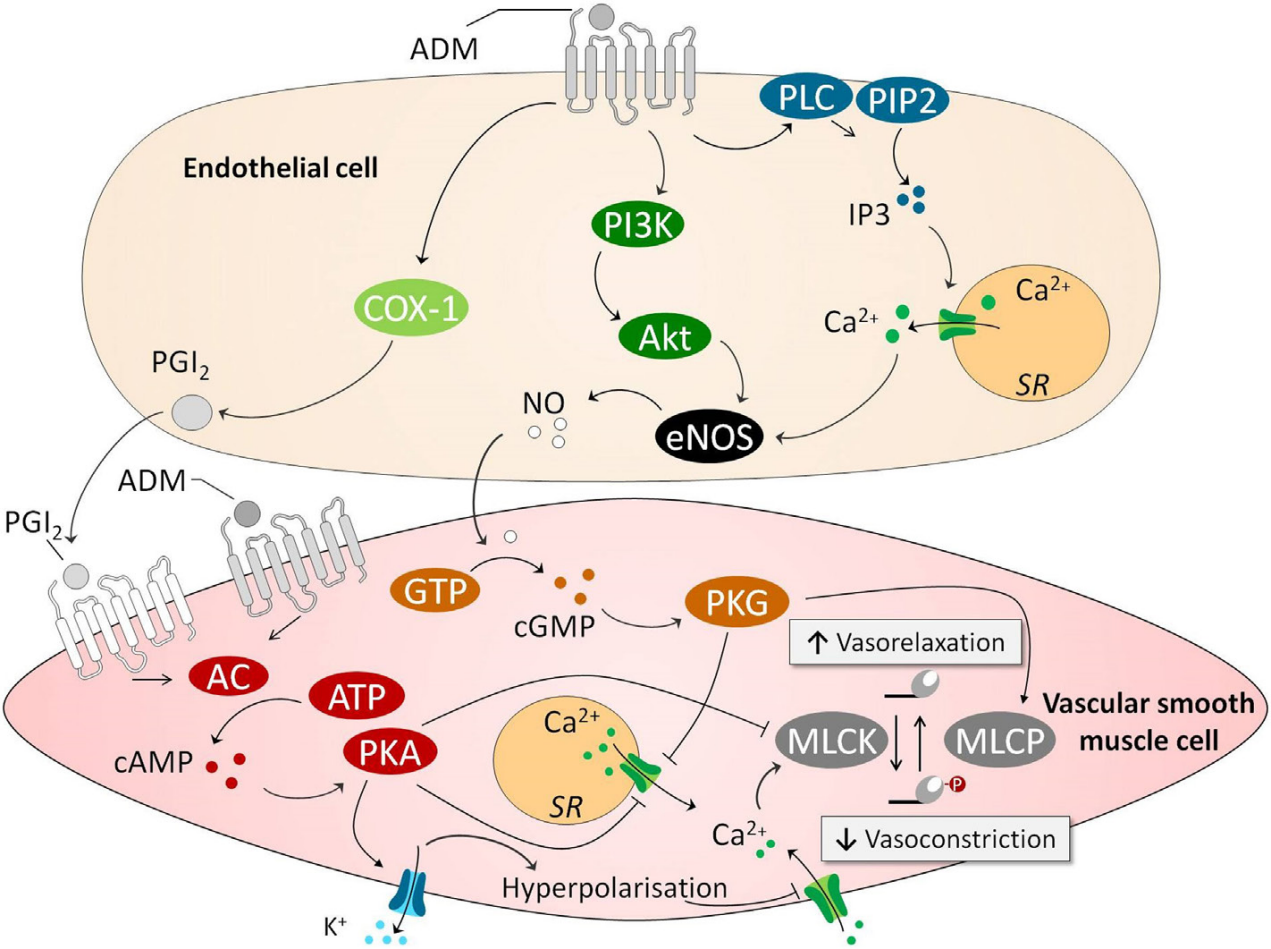
ADM causes vasodilation through endothelium-dependent and endothelium-independent pathways. In an endothelium-independent way, binding of ADM with its receptors on VSMCs increases intracellular cAMP. This leads to subsequent activation PKA, which inhibits smooth muscle cell contraction in several ways. First, PKA opens VSMC potassium channels, causing potassium efflux, leading to membrane potential hyperpolarization and closing of voltage gated calcium channels, reducing intracellular calcium content. Other effects of PKA include inhibition of sarcoplasmatic calcium channel and MLCK. The latter of which is essential for actomyosin contraction. Several endothelium-dependent pathways have been identified. This includes a COX/PGI_2_ pathway that activates the cAMP pathway in VSMCs. Other involved endothelium-dependent pathways are PI3k/Akt and PLC/IP3, which both activate eNOS which leads to subsequently activation of a cGMP/cGMP-dependent kinase pathway in VSMCs. This pathway leads to activation of MLCP which “inactivates” the myosin light chain, and again lowers levels of calcium by inhibiting sarcoplasmatic calcium channels. Abbreviations: AC, adenylyl cyclase; AKT, protein kinase B; ATP, adenosine triphosphate; Ca^2+^, calcium; cAMP, cyclic adenosine monophosphate; cGMP, cyclic guanosine monophosphate; COX-1, cyclooxygenase-1; eNOS; endothelial nitric oxide synthase; GTP, guanosine triphosphate; IP3, inositol triphosphate; MLCK, myosin light chain kinase; MLCP, myosin light chain phosphatase; NO, nitric oxide; PGI_2_, prostacyclin; PI3K, phosphoinositide 3-kinase; PIP2, phosphatidylinositol-4,5-bisphosphate; PKA, protein kinase A; PLC, phospholipase C; SR, sarcoplasmatic reticulum; VSMC, vascular smooth muscle cell; ADM, adrenomedullin.

As mentioned before, the first discovered physiological effect of ADM was vasodilation, leading to hypotension and reduced peripheral resistance (12–14). Over the following years, many studies have confirmed these results. *In vitro* studies demonstrated potent vasodilatory effects of ADM on isolated blood vessels (42, 55) and in isolated organs (56), and *in vivo* studies showed that direct infusion of ADM resulted in decreased blood pressure and induced a compensatory increase of heart rate, endogenous noradrenaline, and renin concentrations in various mammalian species (including humans), which coincided with increased cardiac output (CO) (14, 57–62). These vasodilatory effects of ADM are mediated through binding with its receptors present on vascular ECs and VSMCs (42). It is unknown, although, how both types of interaction contribute quantitatively under physiological and pathophysiological conditions to vasodilation. Several signaling pathways have been described through which ADM causes vasodilation, both endothelium dependent and endothelium independent (55), which are depicted in Figure 1. In an endothelium-independent way, binding of ADM with its receptors on VSMCs increases intracellular cyclic adenosine monophosphate (cAMP) (63, 64), which subsequently activates protein kinase A (PKA, also known as cAMP-dependent kinase) (42). PKA inhibits smooth muscle cell contraction in several ways. For example, it induces the opening of vascular potassium channels, causing potassium efflux, leading to subsequent membrane potential hyperpolarization and closing of voltage gated calcium channels, ultimately reducing intracellular calcium content (42, 65–67). Of note, potassium channel activation is known to play an important role in the blunted norepinephrine responsiveness observed in sepsis (68), and potassium channel blockers have been shown to restore norepinephrine sensitivity in a human *in vivo* model of systemic inflammation (69). Other effects of PKA include inhibition of sarcoplasmatic calcium channels and MLCK. The endothelium-dependent mechanisms through which ADM induces vasodilation are the inositol-1,4,5-triphosphate system and the phosphatidylinositol-4,5-bisphosphate 3-kinase-protein kinase B (PI3K/Akt) pathways. Both of these pathways stimulate endothelial nitric oxide (NO) synthase (eNOS), leading to NO release. In turn, NO activates cyclic guanosine monophosphatase (cGMP) in VSMCs, resulting in activation of protein kinase G, ultimately leading to vasodilation by inhibition of sarcoplasmatic calcium channels and activation of myosin light chain phosphatase (69, 70) and vasodilation. Prostaglandins have also been linked to ADM-induced vasodilation, through the endothelium-dependent cyclooxygenase-1 pathway (42, 71), although results are inconsistent (66), which may be due to differences between animals and the origin of the vessels studied. Finally, it has been suggested that ADM is involved in the central regulation of blood pressure, although these data are equivocal. The presence of ADM has been demonstrated in the hypothalamus (72), and some studies have reported that microinjections of ADM into the hypothalamic paraventricular nucleus elicited a rapid, short decrease in blood pressure (73, 74). Conversely, both infusion of ADM into the intracerebral fluid and microinjections of ADM in the rostral ventrolateral medulla have been shown to increase blood pressure in animal studies (75, 76).

## ADM Regulates Endothelial Barrier Function

The vascular endothelium comprises the inner layer of all blood vessels. This single-cell vascular barrier separates the intravascular from the interstitial space and regulates diffusion of molecules and other substrates through paracellular and transcellular transport (77, 78). Additional roles of the endothelium include regulation of vessel tone, vascular wall permeability, inflammation, hemostasis, and angiogenesis (6, 78, 79). Inflammation leads to barrier compromise at the level of the endothelial cell–cell junction, causing the boundary between intravascular and interstitial spaces to become more porous, subsequently allowing for leakage of inflammatory mediators (e.g., cytokines and prostaglandins) to the interstitium and leukocyte infiltration into the tissues (6). This “leaky barrier” is part of the physiological response to infection, as it is required to combat pathogens in tissues. However, the excessive endothelial barrier disruption observed in sepsis also results in large amounts of fluid leaking from the blood into the tissues, where it accumulates and forms interstitial edema (5, 80). This is a major contributor to the development of shock. Underlying mechanisms of endothelial barrier compromise include rearrangement of the actin cytoskeleton, with cortical actin bundles promoting adherens junction (AJ) formation and EC junction tightening, whereas the formation of stress fibers and phosphorylation of the myosin light chain promotes junction dissociation (81).

Adrenomedullin is essential for endothelial barrier development and barrier stability. In knockout models where crucial parts of the ADM–ADM receptor signaling pathway were deleted, development of lethal hydrops fetalis was noted, indicating inadequate development of the endothelial barrier (82, 83). Moreover, in conditional knockout models, in which either ADM production by ECs, or formation of the RAMP2 part of the ADM1 receptor was abolished, increased vascular permeability and systemic edema formation was observed (84, 85). This coincided with altered expression of the small GTPases Rac1 (Ras-related C3 botulinum toxin substrate 1) and RhoA (Ras homolog gene family, member A), which are involved in the formation of cortical actin and stress fibers; concentration of the protective GTPase Rac1 were reduced whereas levels of the detrimental RhoA GTPase were increased (85).

Additional preclinical work has elucidated underlying intracellular signaling pathways involved in the endothelial barrier-stabilizing effects of ADM. In cultured human umbilical vein endothelial cell cultures and porcine pulmonary artery endothelial cell monolayers, pretreatment with ADM reduced endothelial hyperpermeability elicited by hydrogen peroxide, thrombin, or hemolysin A by attenuating myosin light chain phosphorylation, stress-fiber formation and subsequent gap formation through a cAMP-dependent mechanism (86). Moreover, ADM pretreatment diminished H_2_O_2_-induced edema formation in isolated perfused rabbit lungs, which was accompanied by increased cAMP levels in the lung perfusate (86). Other preclinical work demonstrated similar effects; both treatment with ADM before and following an inflammatory insult reduced endothelial hyperpermeability in *Staphylococcus aureus* α-toxin-exposed isolated rat ileum, again by reducing endothelial myosin light chain phosphorylation and EC contraction (87). In cortactin-deficient HMEC-1 (human microvascular EC) monolayers, which show increased permeability, ADM administration reversed myosin light chain phosphorylation and stress-fiber formation through ADM-induced Rap1 activation and Rock1 inhibition (88). In line, ADM rescued the increase in endothelial permeability in cortactin knockout mice (88). Similar effects were observed in lymphatic ECs, in which ADM stimulation caused a reorganization of the tight junction protein ZO-1 (zonula occludens-1) and VE-cadherin in the plasma membrane, thereby tightening the membrane (89). Other experiments demonstrated barrier disrupting effects of ADM blockade through functional inhibition of the VE-cadherin/β-catenin complex (90). Underlying mechanisms included induction of Src-dependent VE-cadherin phosphorylation, which prevented binding of β-catenin to the cytoplasmic tail of VE-cadherin, inhibiting cell barrier function. Furthermore, β-catenin phosphorylation was induced, which targets β-catenin for ubiquitination and proteasomal degradation. Finally, possible involvement of the PI3K/Akt pathway was suggested (90). These data emphasize that the ADM system is essential for endothelial barrier stabilization.

**Figure 2:**
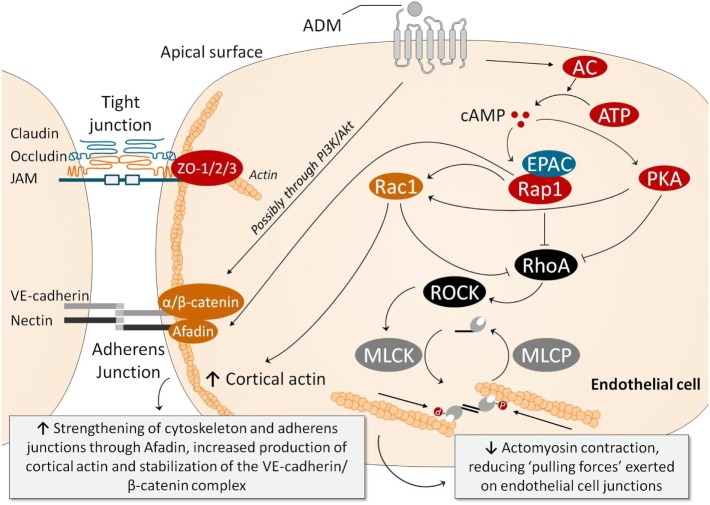
Several pathways have been identified through which ADM exerts endothelial barrier-stabilizing effects. Ligation of ADM with its receptors elicits a strong increase in intracellular cAMP in endothelial cells (ECs), which subsequently activates PKA and, through activation of EPAC, Rap1. PKA and Rap1 inhibit RhoA/ROCK, which results in reduced myosin light chain phosphorylation, decreasing actomyosin contraction (i.e., the “pulling forces” exerted on the EC junctions). Rap1 also promotes junctional adhesiveness *via* Afadin, strengthening junctional tightening by mediating attachment of AJs and the actin cytoskeleton. PKA also increases cortical actin formation through Rac1, which promotes cell–cell stability and cell–matrix adhesion by its connection to tight and AJs. Moreover, Rac1 is also able to inhibit RhoA, decreasing myosin light chain phosphorylation and actomyosin contraction, similar to PKA and Rap1. Ligation of ADM with its receptor is also thought to prevent phosphorylation of VE-cadherin and β-catenin complexes (which would be detrimental for barrier function because phosphorylation of VE-cadherin prevents binding of β-catenin to the cytoplasmic tail of VE-cadherin, and because phosphorylation of β-catenin targets β-catenin for ubiquination and proteasomal degradation), through the PI3K/Akt pathway. Abbreviations: AC, adenylyl cyclase; ADM, adrenomedullin; AJ, adherens junction; ATP, adenosine triphosphate; cAMP, cyclic adenosine monophosphate; cGMP, cyclic guanosine monophosphate; EPAC, exchange factor directly activated by cAMP; MLCK, myosin light chain kinase; MLCP, myosin light chain phosphatase; PI3K/Akt, phosphatidylinositol-4,5-bisphosphate 3-kinase-protein kinase B; PKA, protein kinase A; Rac, Ras-related C3 botulinum toxin substrate 1; Rap1, Ras-related protein-1; ROCK, rho-associated protein kinase; TJ, tight junction; VE-cadherin, vascular endothelial-cadherin; ZO, zonula occludens.

Figure 2 summarizes the mechanisms through which ADM may stabilize the endothelial barrier. Note that the cAMP/PKA pathway once again plays an important role. Ligation of ADM with its receptors elicits a strong increase in intracellular cAMP in ECs, which is thought to be one of the most important signaling molecules involved in stabilization of the endothelial barrier (69, 91). This results in subsequent activation of PKA and inhibition of Rho GTPase (i.e., RhoA; Ras homolog gene family, member A). Independent of PKA, cAMP leads to activation of Rap1 by the Rap1 guanine-exchange factor EPAC (81, 92). Rap1 is thought to enhance EC barrier function in multiple ways, including inhibition of RhoA which reduces actomyosin-induced tension on AJs (81). Moreover, Rap1 promotes junctional adhesiveness *via* Afadin, a promoter of junctional tightening by mediating attachment of AJs and the actin cytoskeleton (81). Finally, both PKA and Rap1 activate Rac1, which results in enforcement of AJs and strengthening of the cortical actin cytoskeleton (93) and inhibition of RhoA (93). Another relevant mechanism through which ADM exerts barrier-enhancing effects is by stabilizing the VE-cadherin/β-catenin complex at the cell–cell junctions, possibly mediated through the PI3K/Akt pathway.

## Miscellaneous Effects of ADM Relevant for Sepsis

The above described data suggest potential utilization of the ADM system for the treatment of diseases with marked endothelial barrier dysfunction, of which septic shock is a prime example, although these beneficial properties might be offset by vasodilatory effects, an issue we will discuss later on in this review. Furthermore, next to effects on vascular tone and the endothelial barrier, ADM also has other properties relevant in the context of sepsis, including immunoregulatory, antimicrobial and cardioprotective effects.

### Immunoregulatory Effects

**Figure 3:**
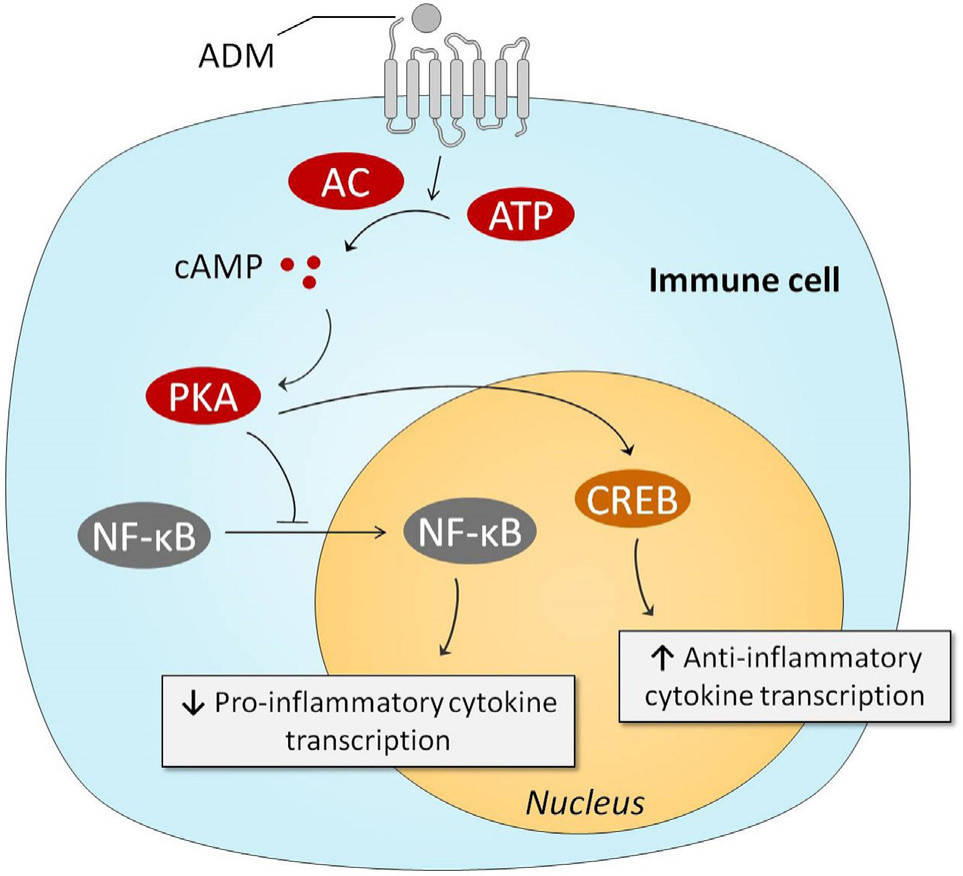
Intracellular mechanisms behind ADM-induced anti-inflammatory effects. Stimulation of the ADM receptors results in increased intracellular cAMP concentrations, which subsequently activate PKA. PKA prevents NF-κB from entering the nucleus, resulting in reduced transcription of pro-inflammatory genes. PKA-induced activation of CREB results in augmented anti-inflammatory transcription of anti-inflammatory cytokines. Abbreviations: AC, adenylyl cyclase; ADM, adrenomedullin; ATP, adenosine triphosphate; cAMP, cyclic adenosine monophosphate; CREB, cAMP response element-binding protein; NF-κB, nuclear factor kappa-light-chain-enhancer of activated B cells; PKA, protein kinase A.

The immune system plays a pivotal role in the pathogenesis of sepsis (4, 8). Therefore, it is relevant to discuss the potential immunoregulatory effects of ADM. Several *in vitro* studies have demonstrated that ADM exerts anti-inflammatory effects, and we have summarized the involved pathways in Figure 3. One of the first studies conducted on this matter, investigated the effects of ADM in lipopolysaccharide (LPS) stimulated rat alveolar macrophages. Interestingly, ADM significantly inhibited cytokine-induced neutrophil chemoattractant (CINC/CXCL-1) release, possibly through a cAMP-dependent mechanism (94). Other experiments in Swiss 3T3 murine fibroblasts demonstrated that ADM inhibits interleukin-1 beta-induced tumor necrosis factor alpha (TNFα) secretion and confirmed the major role of the cAMP–PKA pathway: a cAMP-dependent protein kinase inhibitor was able to negate ADMs inhibitory effects (95). Similar effects of ADM were observed in microglia upon stimulation with LPS, inhibiting both TNFα and interleukin (IL)-6 (96), as well as in LPS-stimulated murine RAW264.7 macrophages and rat Kupffer cells (97). *In vivo* experiments have confirmed these *in vitro* studies. Coadministration of ADM and ADM-binding protein-1 (AMBP-1) (a protective peptide with putative ADM-enhancing effects) in a rat endotoxemia model attenuated the TNFα response through a mechanism that involves peroxisome proliferator-activated receptor-gamma (98). Interestingly, ADM has also been a subject of interest for the treatment of inflammatory bowel disease. Intracolonic administration of ADM resulted in a dose dependent and significant reduction of the size of the ulcerative lesions in a model of acetic acid-induced colitis, and reduced tissue IL-6 levels (99). Subsequent studies have confirmed these results. For instance, lower levels of interferon-γ (IFN-γ) and TNFα were observed in rodent models of dextran sulfate sodium-induced colitis (100, 101). A case series on seven ulcerative colitis patients that received intravenous infusion of ADM for 8 h daily over a period of 2 weeks reported improved disease activity index scores, and substantial improvement of ulcers upon endoscopic examination (102). No serious adverse effects were observed, apart from minor effects on blood pressure and heart rate.

### Antimicrobial Properties

The epithelium represents the first protective barrier against pathogens. Many types of epithelial cells secrete ADM, and it can thus be found in many bodily fluids at much higher concentrations than in plasma (103, 104). ADM has chemical and structural similarities with other antimicrobial peptides (i.e., β-defensin-2), including peptide length, a net positive charge, a disulfide bond between residues 16 and 21 and an amidated tyrosine at the carboxyl terminus (105). This forms an amphipathic structure, which permits bacterial membrane intercalation (106). *In vitro* studies have demonstrated that both the ADM peptide and also smaller ADM fragments are able to inhibit bacterial growth (107).

### Cardiac Protection

Adrenomedullin may also confer cardioprotective effects. Increased cardiac hypertrophy and fibrosis were observed after subjecting heterozygous ADM knockout mice to stress-induced cardiac hypertrophy compared with their wild-type counterparts (108, 109). Other work demonstrated ADM-induced reduction of doxorubicin-induced cardiac myocyte apoptosis *via* a cAMP-dependent mechanism (110), which was later confirmed *in vivo* in a mice model (111). The first steps concerning ADM treatment in heart failure patients have been undertaken. In patients with stable congestive heart failure, a short-course ADM infusion resulted in a significant decrease of pulmonary capillary wedge pressure and pulmonary arterial pressure, as well as an increase of cardiac index (58). Moreover, ADM increased urinary volume and sodium excretion, while decreasing plasma aldosterone levels. In a pilot study in patients with acute decompensated heart failure, combined therapy of ADM and human atrial natriuretic peptide also resulted in beneficial hemodynamic and hormonal changes, including decreased pulmonary arterial pressure, increased urine production and reduced aldosterone and brain natriuretic peptide plasma concentrations (112). Until now, no further studies have been conducted in patients with heart failure.

In contrast to the data presented earlier, ADM has also been named a “cardiac depressant factor,” because administration of an ADM-receptor antagonist resulted in increased myocyte contractility in isolated ventricular cardiac myocytes during the early phase rat endotoxemia, although no measurements of CO were performed (113, 114).

## ADM in Sepsis

Several processes that take place during sepsis stimulate ADM secretion, including hypoxia, increased circulating levels of LPS, and production of cytokines such as tumor necrosis factor, interleukin-1, and IFN-γ (33, 115–118). Circulating ADM levels have been measured in various pathophysiological conditions, and interestingly, the highest concentrations were found in patients with septic shock (119–122). In sepsis patients, circulating ADM levels correlated with relaxation of vascular tone (123) as well as with disease severity and mortality (119–121, 124). These associations suggest that ADM may play a detrimental role in sepsis, and that ADM-targeted therapies could be of benefit. However, no causal relationships can be deducted from these observational studies, and it may also be possible that increases in ADM represent a (failing) compensatory response. In other words, in light of ADMs aforementioned beneficial effects on various pathophysiological processes that take place during sepsis, increased ADM levels might also represent a strategy employed by the body to curtail organ damage during sepsis.

## ADM and ADM-Targeted Therapy as Treatment Strategies Relevant for Sepsis

Over the last decades, many have sought to investigate whether administration of ADM, modulation of its function, or antagonizing ADM may influence outcome in various preclinical models of sepsis as well as in models of systemic inflammation and organ injury. Below, we provide an overview of the available data on each of these treatment strategies. Please note that models of systemic inflammation and organ injury do not comprehensively mimic sepsis, but do capture distinct pathophysiological hallmarks of the disease and are therefore of relevance for this overview.

### ADM Administration

An overview of preclinical studies that have investigated the effects of ADM administration is presented in Table 1. It needs to be emphasized that except for one, these studies were not performed using infection models, but in clinically less relevant models of systemic inflammation or organ injury.

**Table 1:** Overview of preclinical studies investigating ADM administration in different models related to sepsis.

Intervention	Model	Results (compared with placebo)	Reference
Bolus of ADM (pretreatment)	H_2_O_2_-induced vascular leakage in isolated, mechanically ventilated rabbit lungs	↓ Vascular leakage	Hippenstiel et al. (86)

Continuous infusion of incremental dosages of ADM (posttreatment)	Ovine endotoxemia (24 h of *Salmonella typhosa* LPS administration)	↓ Pulmonary vascular resistance ↑ Cardiac index and heart rate	Westphal et al. (128)

Continuous infusion of ADM (pre- and posttreatment groups)	Isolated rat ileum with *Staphylococcus aureus* α-toxin administration	↑ Endothelial barrier function in both pre- and posttreatment groups.	Brell et al. (87)

Continuous infusion of ADM (pre- and posttreatment groups)	Ovine endotoxemia (4 h of *Salmonella typhosa* LPS administration)	↑ Cardiac index ↓ Pulmonary arterial pressure ↑ Lactate clearance (for both pre- and posttreatment groups)	Ertmer et al. (125)

Continuous infusion of ADM (pretreatment)	Intratracheal LPS-induced lung injury in rats	↓ Vascular leakage ↓ Histopathological abnormalities ↓ Protein, albumin, and inflammatory markers in BAL fluid	Itoh et al. (126)

Continuous infusion of ADM (posttreatment)	*S. aureus* α-toxin model in resuscitated rats	↓ Vascular leakage ↑ 6-h blood pressure ↑ Cardiac index and 6-h survival	Temmesfeld-Wollbrück et al. (127)

Continuous infusion of ADM (posttreatment)	*S. aureus* α-toxin model in rats	↓ Gut epithelial hyperpermeability	Temmesfeld-Wollbrück et al. (129)

Continuous infusion of ADM (started pretreatment in 2 h experiments, and after 2 h of ventilation in the 6 h experiments)	Ventilator-induced lung injury model in mice, experiments with 2 and 6 h of ventilation	For both experiments ↓ Lung hypermeability, leukocyte accumulation, and MLCP expression ↑ Oxygenation, lactate, and creatinine clearance	Müller et al. (130)

Bolus of intrapleural ADM (posttreatment)	Carrageenan-induced pleurisy model in mice	↓ Pro-inflammatory cytokines ↓ Oxidative and nitroxidative lung tissue injury	Talero et al. (132)

Continuous infusion of ADM (pretreatment)	Aortic ischemia–reperfusion in rats	↓ Kidney injury (various morphological and biochemical parameters)	Oyar et al. (134)

Bolus of ADM (pretreatment)	Contrast-induced nephropathy in rats	↓ Kidney injury and inflammation	Inal et al. (133)

Continuous infusion of ADM (pretreatment)	Pneumococcal pneumonia in mechanically ventilated mice	↓ Lung injury ↓ Lung hyperpermeability ↓ Indirect liver and gut injury	Müller-Redetzky et al. (131)

*ADM, adrenomedullin; BAL, bronchoalveolar lavage; H_2_O_2_, hydrogen peroxide; LPS, lipopolysaccharide; MLCP, myosin light chain phosphatase*.

*Only studies using models of sepsis or models that capture some of the prominent hallmarks of sepsis have been included*.

Adrenomedullin administration resulted in improved hemodynamics, reduced vascular leakage and organ damage, and improved outcome in different models of endotoxemia (125–129). Furthermore, beneficial effects of ADM were reported on various outcome measurements in models of lung injury, including attenuated endothelial hyperpermeability, liver injury, less histopathological changes, and reduced pro-inflammatory cytokine levels (130–132). Moreover, ADM showed protective effects on organ injury in several models of acute kidney injury (133, 134). Although the potential beneficial effects of ADM infusion have been extensively investigated in the abovementioned models of endotoxemia, lung- and renal injury, data obtained in models that are more relevant to sepsis (for example, in resuscitated cecal ligation and puncture [CLP] models in larger animals) are lacking.

There may be some drawbacks to ADM administration. Because of the short half-life of ADM (22 min) (47), infusion would have to be continuous over longer periods of time, as was done previously in ulcerative colitis patients (102). Moreover, as alluded to before, ADM has potent vasodilatory effects, which may raise concerns for ADM-induced hypotension. Finally, ADM may be difficult to handle in clinical practice, because of its adhesiveness, arguably sticking to artificial surfaces (135).

### Coadministration of ADM and Complement Factor H

Complement factor H is thought to be capable of binding to ADM (and therefore also known as AMBP-1 in this context), and chaperone ADM in the circulation (136). However, note that this has been subject to some debate in literature. The observed *in vitro* binding could theoretically be due to unspecific (ionic) interaction. Interference of complement factor H with ADM could not be demonstrated in other recent work, in which up to almost 400,000-fold molar excess of complement factor H did not influence ADM recovery (137). Furthermore, *in vivo* plasma levels of complement factor H are approximately 10^9^-fold higher than ADM levels. Therefore, it could be hypothesized that exogenous administered complement factor H would not add significantly to endogenous levels.

The ADM binding site of AMBP-1 has not yet been discovered, although it is thought that AMBP-1 may modulate ADM activity and degradation. A functional assay revealed a twofold increased cAMP response after coincubation of cells with ADM and AMBP-1 compared with incubation with ADM alone (138). Other work has demonstrated that AMBP-1 protects ADM from proteolytic degradation (49), thereby presumably increasing its half-life. Because of these possible potentiating effects, several studies have investigated the therapeutic potential of coadministration of ADM and AMBP-1 in various preclinical animal models. Initially, the effects of coadministration of ADM and AMBP-1 were assessed in a model of CLP-induced sepsis in rats, where pretreatment with the combination of ADM and AMBP-1, but not of each compound individually, resulted in positive effects on hemodynamic parameters, augmenting oxygen delivery, CO, and lactate clearance (139). Furthermore, improved 10-day survival was observed in animals undergoing CLP surgery. Note that in these survival experiments, treatment was started 5 h after CLP surgery. Other studies have also demonstrated beneficial effects coadministration of ADM and AMBP-1 in models of hemorrhagic shock (140–142), ischemia/reperfusion (143, 144), endotoxemia (98), and septic shock (145, 146). To what extent complement factor H influences the described ADM-mediated effects remains unclear as the majority of preclinical studies on ADM with AMBP-1 coadministration did not compare ADM/AMBP-1 with ADM alone. Please refer to Table 2 for an overview of this work.

**Table 2:** Overview of preclinical studies investigating ADM with coadministration of AMBP-1 in different models related to sepsis.

Intervention	Model	Results (compared with placebo)	Reference
ADM and AMBP-1 (posttreatment)	Cecal ligation and puncture (CLP) induced sepsis in rats	↑ CO, DO_2_, and lactate clearance ↑ Hepatic blood flow ↓ Plasma ALT, AST ↓ Hemodilution ↑ 10-day survival	Yang et al. (139)
ADM and AMBP-1 for 45 min (posttreatment)	Hemorrhagic shock rats (MAP 40 mmHg for 90 min), then resuscitated	↓ Plasma AST, ALT, TNF, and HMGB-1 ↑ IL-10 ↑ Lactate and creatinine clearance ↑ 12-day survival	Cui et al. (140)
ADM and AMBP-1 (posttreatment)	Hemorrhagic shock rats (MAP 40 mmHg for 90 min), then resuscitated	↑ CO and organ blood flow (liver, kidney, and small intestine) ↓ Cardiac TNF-α	Wu et al. (142)
ADM and AMBP-1 at start of reperfusion	Intestinal ischemia–reperfusion in rats	↓ Plasma TNF-α, IL-1β, IL-6, and IL-10 ↓ Plasma AST and ALAT ↓ Histopathological changes small intestine ↑ Lactate and creatinine clearance ↑ 10-day survival	Carrizo et al. (144)
ADM and AMBP-1 (pretreatment)	Endotoxemic rats	↓ TNF-α ↑ IL-10 ↑ Lactate clearance	Miksa et al. (98)
ADM and AMBP-1 (pretreatment)	CLP-induced sepsis in rats	↑ eNOS signaling ↓ Endothelial dysfunction	Zhou et al. (156)
ADM and AMBP-1 at start of reperfusion	Intestinal ischemia–reperfusion-induced lung injury in rats	↓ Lung edema ↓ Lung TNFα and IL-6 ↓ Histopathological changes	Dwivedi et al. (143)
ADM and AMBP-1 (posttreatment)	Hemorrhagic rats (MAP 40 mmHg for 90 min), then resuscitated	↓ Plasma AST and ALT ↑ Lactate and creatinine clearance ↓ Plasma TNF-α and IL-6 ↑ 12-day survival	Wu et al. (141)
ADM and AMBP-1 at start of reperfusion	Renal ischemia–reperfusion in rats	↓ Renal edema ↓ Tissue injury ↓ Plasma and tissue pro-inflammatory cytokines	Shah et al. (157)
ADM and AMBP-1 (posttreatment)	Bile duct ligation/CLP model of induced sepsis in rats	↓ Systemic markers of tissue injury ↓ Inflammatory response ↑ 7-day survival	Yang et al. (146)

*ADM, adrenomedullin; ALT, alanine aminotransferase; AMBP-1, ADM-binding protein-1; AST, aspartate aminotransferase; BAL, bronchoalveolar lavage; CLP, cecal ligation and puncture; CO, cardiac output; DO_2_, delivery of oxygen rate; H_2_O_2_, hydrogen peroxide; HMGB-1, high mobility group box protein-1; IL, interleukin; LPS, lipopolysaccharide; TNF, tumor necrosis factor; TNFα, tumor necrosis factor alpha; IL-1β, interleukin-1 beta*.

*Only studies using models of sepsis or models that capture some of the prominent hallmarks of sepsis have been included*.

### Antibodies against and/or Receptor Antagonists of ADM

To date, three studies have investigated the effects of ADM antagonists on hemodynamic parameters in preclinical models of endotoxemia and sepsis, using either a neutralizing anti-ADM antibody (147) or the ADM receptor antagonist ADM (22–52) (113, 148). Both these treatments prevented the occurrence of a “hyperdynamic” hemodynamic response (characterized by decreased blood pressure and peripheral vascular resistance, and an increased CO) during the first hours after induction of sepsis or systemic inflammation (147, 148). This is in line with previous data demonstrating vasodilatory effects of ADM, accompanied by a reduction of peripheral vascular resistance and increase of CO. Furthermore, ADM (22–52) administration after initiation of endotoxemia resulted in improved myocyte contractility, but did not improve 7-day survival (113).

Further efforts have been put into the development of various high-affinity monoclonal anti-ADM antibodies, each targeting different regions of the ADM peptide, resulting in full or partial inhibition of ADM signaling. The efficacy of these antibodies was investigated in a survival study in CLP-induced sepsis in mice (149). A non-neutralizing antibody targeted against the N-terminus of ADM, which only partially inhibits ADM signaling, conferred survival benefit, whereas a completely inhibiting antibody targeted against the C-terminal, did not. Subsequent experiments were conducted in a model of resuscitated CLP-induced murine sepsis, in which pretreatment with the non-neutralizing antibody resulted in decreased catecholamine infusion rates, kidney dysfunction, iNOS, but not eNOS expression, and ultimately improved survival (150). Due to these positive results, a humanized version of the antibody, named Adrecizumab, has been developed for further clinical development. Beneficial effects of Adrecizumab on vascular barrier function and survival were recently demonstrated in preclinical models of systemic inflammation and sepsis (151). In this study, pretreatment with Adrecizumab attenuated renal vascular leakage in endotoxemic rats as well as in mice with CLP-induced sepsis, which coincided with increased renal expression of the protective peptide Ang-1 and reduced expression of the detrimental peptide vascular endothelial growth factor (151). Also, pretreatment with Adrecizumab improved 7-day survival in CLP-induced sepsis in mice from 10 to 50% for single and from 0 to 40% for repeated dose administration (151). Moreover, in a phase I study, excellent safety and tolerability was demonstrated: no serious adverse events were observed, no signal of adverse events occurring more frequently in Adrecizumab-treated subjects was detected, and no relevant changes in other safety parameters were found (152). Of particular interest is the proposed mechanism of action of Adrecizumab. Both animal and human data reveal a potent, dose-dependent increase of circulating ADM following administration of this antibody. Based on pharmacokinetic data and the lack of an increase in MR-proADM (an inactive peptide fragment derived from the same prohormone as ADM), the higher circulating ADM levels cannot be explained by an increased production (152). A mechanistic explanation for this increase could be that the excess of antibody in the circulation may drain ADM from the interstitium to the circulation, since ADM is small enough to cross the endothelial barrier, whereas the antibody is not. In addition, binding of the antibody to ADM leads to a prolongation of ADM’s half-life (153). Even though Adrecizumab partially inhibits ADM signaling, a large increase of circulating ADM results in an overall “net” increase of ADM activity in the blood compartment, where it exerts beneficial effects on ECs (predominantly barrier stabilization), whereas ADMs detrimental effects on VSMCs (vasodilation) in the interstitium are reduced (153). This hypothesis is in line with previous studies demonstrating overall beneficial effects of agonists of the ADM system, whereas complete inhibition of ADM was shown not to improve outcome. A detailed description of the proposed mechanisms of action of Adrecizumab is provided elsewhere (153). Please refer to Table 3 for an overview of studies that investigated ADM-antagonists and/or modulating antibodies. Currently, a phase II study with Adrecizumab is ongoing in septic patients (http://clinicaltrials.gov identifier: NCT03085758).

**Table 3:** Overview of preclinical studies with ADM antibodies and/or antagonists in different models related to sepsis.

Intervention	Model	Results	Reference
Anti-ADM antibody (posttreatment)	CLP-induced sepsis in rats	Anti-ADM antibodies prevented occurrence of hyperdynamic response during first 5 h after CLP	Wang et al. (147)
ADM receptor antagonist ADM (22–52) (pretreatment)	*Escherichia coli* LPS in rats	↑ Blood pressure (during first 6 h)	Mazzocchi et al. (148)
ADM antagonist ADM (22–52) (posttreatment)	*E. coli* LPS in rats	↑ Survival myocyte contractility No effect on 7-day survival	Hyvelin et al. (113)
N-terminus murine antibody against N-terminus of ADM (pretreatment)	CLP-induced sepsis in mice	↑ Survival	Struck et al. (149)
N-terminus murine antibody against N-terminus of ADM (pretreatment)	Resuscitated CLP-induced sepsis in mice	↓ Noradrenaline infusion rates ↑ Urine production ↑ Creatinine clearance and ↓ NGAL ↓ iNOS and peroxynitrate formation ↓ Systemic inflammation ↓ Tissue apoptosis	Wagner et al. (150)
N-terminus humanized antibody against N-terminus of ADM (pretreatment)	*E. coli* LPS in ratsCLP-induced sepsis in mice	↓ Vascular leakage in LPS rats ↓ Renal vascular leakage, ↓ VEGF, and ↑ angiopoietin-1 levels in CLP mice ↑ Survival in CLP mice	Geven et al. (151)

*ADM, adrenomedullin; CLP, cecal ligation and puncture; LPS, lipopolysaccharide; VEGF, vascular endothelial growth factor; eNOS; endothelial nitric oxide synthase*.

*Only studies using models of sepsis or models that capture some of the prominent hallmarks of sepsis have been included*.

### PEGylation of ADM

PEGylation is the process by which polyethylene glycol (PEG) chains are attached to protein and peptide drugs (154). PEGylation of polypeptide drugs often results in improved pharmacokinetic and pharmacodynamic properties, as it offers protection from proteolytic enzymes, increases water solubility, reduces renal clearance, and limits toxicity (154). Human ADM was previously molecularly modified by conjugating ADMs N-terminal with PEG, in an attempt to reduce potentially unfavorable effects of ADM (hypotension, activated sympathetic nerve activity, and increased renin secretion) (155). Compared with native ADM, PEGylated ADM had a slightly lower half maximal effective concentration (EC_50_) in a functional assay, while the maximum possible effect (*E*_max_) values remained similar. Moreover, in rats, PEGylated ADM resulted in a longer half-life and a significantly less blood lowering effect compared with native ADM (155). A subsequent study in a mouse DSS-induced colitis model revealed an attenuation of the total inflammation score. Unfortunately, no studies have been performed in animal sepsis models.

## Conclusion

Adrenomedullin is an important peptide hormone involved in sepsis. Its effects include vasodilation, stabilization of the endothelial barrier, and immunoregulation. Administration of ADM in animal models of inflammation, organ injury, and infection resulted in improved outcome. Attempts have been made to negate the potential hypotensive effects of ADM to further enhance its beneficial effects. Coadministration of ADM with ADM binding peptide-1, administration of ADM bound to PEG, and administration of partially inhibiting ADM antibodies (which in fact increase the net circulating ADM levels without causing hypotension) showed promising results. However, it is difficult to translate these results to septic patients, because these preclinical studies have often been performed in small animals using clinically less relevant models of systemic inflammation or induced organ injury. Moreover, in a significant proportion of studies no resuscitation or antibiotics were applied, and the intervention was initiated before the induction of disease. Finally, many treatments have not been compared head-to-head. Given the current lack of adjuvant therapies in sepsis, future research on this promising peptide in more relevant animal models of sepsis and ultimately humans is therefore highly warranted.

## Author Contributions

CG wrote the manuscript. MK and PP supervised the writing and critically reviewed the manuscript.

## Conflict of Interest Statement

PP’s institution has received a research grant from Adrenomed AG (patent owner of adrenomedullin antibodies). PP received travel reimbursements and consultancy fees from Adrenomed AG. All other authors declare that the research was conducted in the absence of any commercial or financial relationships that could be construed as a potential conflict of interest.
